# The influence of personality traits on college students’ exercise behavior: a chain mediation model of exercise self-efficacy and exercise motivation

**DOI:** 10.1186/s40359-025-03220-y

**Published:** 2025-08-04

**Authors:** Qishun Yang, Mingliang Song, Min Li, Xiao Chen

**Affiliations:** 1 School of Physical Education and National Equestrian, Wuhan Business University, Wuhan, China; 2 Research Center for Modern Equine Industry Development, Wuhan Business University, Wuhan, China; 3 School of Football, Wuhan Sports University, Wuhan, China; 4 School of General Education, Hunan University of Information Technology, Changsha, China; 5 School of Sports Training, Tianjin University of Sport, Tianjin, China; 6 Phsycial Education Department, Zhongnan University of Economics and Law, Wuhan, China

**Keywords:** Personality traits, Self-efficacy, Motivation, Exercise behavior, Chain mediation model, College students

## Abstract

**Purpose:**

Previous studies have shown that personality traits have varying degrees of association with exercise behavior. However, the underlying mechanisms mediating this relationship remain insufficiently understood. This study aims to investigate the associations between the Big Five personality traits and exercise behavior and to construct a chain mediation model incorporating exercise self-efficacy and exercise motivation, based on the Cognitive-Affective Personality System theory, Social cognitive theory, and Self-Determination theory with the practical goal of providing guidance for promoting physical well-being among college students.

**Methods:**

A total of 483 non-physical education majors undergraduate students (mean age = 19.71 ± 1.31 years, 56.1% female) from five randomly selected universities in Wuhan participated in the study. Data were collected through electronic questionnaires administered at two time points: baseline (Time 1; T1) assessing demographics, personality traits, and exercise self-efficacy, and four weeks later (Time 2; T2) assessing exercise motivation and behavior. The data were analyzed using SPSS 26.0 and the PROCESS macro, employing correlation and chain mediation analyses to explore the relationships among variables.

**Results:**

The findings revealed that Openness, Conscientiousness, and Extraversion were positively associated with exercise behavior, Neuroticism was negatively associated, and Agreeableness showed no significant association. Mediation analysis indicated that Conscientiousness, Extraversion, and Neuroticism were related to exercise behavior through exercise self-efficacy, whereas Openness, Conscientiousness, Extraversion, and Neuroticism were related to exercise behavior through exercise motivation. Additionally, a significant chain mediation effect was observed for Conscientiousness, Extraversion, and Neuroticism, wherein exercise self-efficacy and exercise motivation sequentially mediated the relationship between these personality traits and exercise behavior. Notably, Openness did not significantly relate to exercise self-efficacy, suggesting that its relationship with exercise behavior was solely mediated by exercise motivation. Furthermore, significant gender differences were found in exercise motivation, with males exhibiting higher exercise motivation than females.

**Conclusion:**

This study elucidates how personality traits are associated with college students’ exercise behavior through the mediating roles of exercise self-efficacy and exercise motivation. Specifically, Extraversion and Neuroticism demonstrated partial mediation through both self-efficacy and motivation, while Openness was associated with exercise behavior solely via motivation. The association between Conscientiousness and exercise behavior was fully mediated by both factors, indicating a complete indirect pathway. Although directly changing personality traits is challenging, targeting modifiable psychological mechanisms—such as enhancing self-efficacy and intrinsic motivation—may effectively promote exercise engagement. Personalized intervention strategies that account for individual personality characteristics and gender-specific motivational factors are recommended to support students’ physical and mental health and reduce long-term health risks.

**Supplementary Information:**

The online version contains supplementary material available at 10.1186/s40359-025-03220-y.

## Introduction

The fitness of college students has been a longstanding concern for both the Chinese government and scholars [1, 2]. The concept of ‘strong youth, strong China; strong sports, strong China’ underscores the integral role of advancing the development of China’s sports in the nation’s great rejuvenation [3]. The physical health of adolescents, as successors in national, economic, and cultural development, determines the foundation of their overall well-being in the future. However, globally, over 80% of students lack sufficient physical activity, with college students comprising a significant portion of this demographic [4–6]. This issue is particularly pressing in China, where the Eighth National Survey on Students’ Physical Fitness reports a decline in college students’ physical health [7, 8].

The lack of physical activity is a major cause of poor physical fitness among college students [2] and insufficient activity is associated with numerous health risks, including obesity, type-2 diabetes [9] hypertension [10] and mental health disorders [11]. Many Chinese college students experience depression, social anxiety, sleep difficulties, and even suicidal tendencies [12]. Given these challenges, cultivating good exercise behavior among college students is essential [13]. exercise behavior refers to the patterns, frequency, and types of physical activities that individuals engage in to improve or maintain their physical health, fitness, and overall well-being [14]. It encompasses not only the extent of individuals’ participation in physical activity but also how they plan and organize their exercise routines [15]. Good exercise behavior can guide students to establish healthy lifestyles, improve physical fitness, and prevent the development of psychological problems [16]. For instance, Kayani et al. found that regular aerobic exercise significantly reduces symptoms of depression and anxiety among college students [17]. Similarly, Ma et al. demonstrated that increased physical activity is associated with improved sleep quality and reduced stress levels in young adults [18]. In addition, developing good exercise habits and engaging in regular physical activity during college years is crucial for students’ long-term well-being and career productivity [19].

One significant factor that influences exercise behavior is individual personality traits [20] which are recognized as relatively stable patterns of thoughts, emotions, and behaviors [21]. These traits not only reflect an individual’s tendency to respond to the environment but also predict their potential health behavior choices [22]. Personality traits are closely linked to emotional experiences and emotion regulation. Diotaiuti et al. emphasize that emotional balance and self-regulation play critical roles in academic and behavioral performance, highlighting them as essential constructs in analyzing how personality influences behavior [23].

The Big Five personality traits (Openness, Conscientiousness, Extraversion, Agreeableness, and Neuroticism) are widely used to assess personality dimensions [24]. Openness refers to an individual’s willingness to engage with new experiences and ideas. It encompasses characteristics like imagination, creativity, intellectual curiosity, and a preference for novelty and variety. Conscientiousness reflects a person’s degree of organization, responsibility, and dependability. Traits include being disciplined, goal-oriented, and having a strong sense of duty. Extraversion measures the extent of an individual’s interpersonal interactions, activity level, seeking stimulation, and capacity for enjoyment. Agreeableness assesses whether an individual tends to orient themselves toward sympathy or antagonism in terms of thoughts, emotions, and actions within the interpersonal domain. Neuroticism is used to gauge the degree of emotional stability in individuals, indicating a predisposition toward experiencing negative emotions. This dimension includes tendencies such as anxiety, depression, anger, impulsivity, sensitivity, and vulnerability [24–26]. Accurate and culturally relevant personality assessment has become a crucial aspect of sports psychology to ensure valid research findings and effective application [27, 28].

Empirical studies have demonstrated significant associations between certain personality traits and exercise behavior. For instance, conscientious individuals are more likely to formulate and follow exercise plans due to their disciplined nature [29]. Extraverted individuals, being sociable and energetic, may be more inclined to engage in group sports and physical activities [20]. Conversely, individuals with higher levels of Neuroticism may avoid physical activity due to anxiety or fear of negative outcomes [30, 31]. While Openness has been less studied, its association with a preference for new experiences suggests a potential positive relationship with exercise behavior [20, 32]. As for Agreeableness, previous studies have generally reported non-significant or inconsistent findings regarding its relationship with exercise behavior [33–35]. Nevertheless, we included all five personality traits in our analysis to examine whether these relationship hold in our sample.

Most previous studies have focused on associations between personality traits and exercise behavior, with limited research exploring potential mediating pathways that may link traits to behavior. Acknowledging that personality traits are stable characteristics not amenable to experimental manipulation, we aimed to examine how these traits might be related to exercise behavior through theoretically grounded cognitive and affective processes. To explore these mechanisms, theoretical frameworks that consider such processes are necessary. One such framework is the Cognitive-Affective Personality System Theory (CAPS) [36, 37] which suggests that behavior is not solely the result of stable personality traits but also involves the processing of environmental information, as well as beliefs, expectations, and emotions toward external stimuli [38]. Personality traits, as stable characteristics, may influence cognition (self-efficacy and expectancies) [36] emotions, and behaviors [39, 40]. Therefore, we included exercise self-efficacy and exercise motivation in our study as potential mediators.

Self-efficacy, as a central component of Bandura’s social cognitive theory (SCT), has been consistently recognized as a key determinant of various health behaviors, including physical activity [41, 42]. Exercise self-efficacy refers to specific manifestation of self-efficacy within the context of physical activity, which encapsulates an individual’s belief in their ability to successfully engage in physical behaviors. This belief influences the individual’s choice of tasks, effort, persistence, and response to challenges, and plays a crucial role in determining the intensity and regularity of physical activity participation [43, 44]. Higher self-efficacy enables individuals to manage and regulate negative emotional experiences during physical challenges, fostering an emotionally stable disposition and increasing persistence in exercise [45]. Previous studies have confirmed that college students’ exercise behavior is positively associated with their exercise self-efficacy [46, 47]. In addition, a cross-sectional study discovered a relationship between exercise behavior and self-efficacy in Spanish adolescents, advocating for the development of self-efficacy as a strategy for adolescent exercise behavior [48]. Hou et al. indicated that self-efficacy has a moderating influence on the association between exercise intentions and behaviors, facilitating the transition from exercise intentions to actual behaviors [49].

Although research on the relationship between exercise self-efficacy and personality traits is currently limited, integrating the Big Five model and SCT allows us to infer that personality traits influence an individual’s self-efficacy, which in turn affects their exercise behavior [41, 50]. Specifically, Individuals with higher levels of Extraversion tend to have greater self-efficacy [51]. Individuals with higher levels of Conscientiousness, characterized by discipline and goal orientation, are likely to have higher self-efficacy because they believe in their ability to follow through with exercise plans [52, 53]. Conversely, individuals with higher level of Neuroticism are more prone to report negative self-efficacy, meaning they tend to have lower confidence in their ability to engage in physical activities [54]. However, the relationship between Openness and self-efficacy has shown inconsistent results across different studies [53, 55]. Therefore, based on these theoretical frameworks and previous research, exercise self-efficacy may act as a cognitive mechanism that mediates the relationship between personality traits and exercise behavior in Chinese college students, with varying degrees.

In addition to exercise self-efficacy, exercise motivation is another critical factor that may mediate the relationship between personality traits and exercise behavior. Motivation refers to the psychological drive that compels individuals to engage in physical activity, encompassing both intrinsic factors (e.g., enjoyment, competence development) and extrinsic factors (e.g., health benefits, social interactions) [56]. Self-Determination Theory (SDT) emphasizes the importance of motivation in promoting behaviors [57] classifying it along a continuum from controlled motivation to autonomous motivation [58]. Rooted in SDT, exercise motivation extends this theoretical framework by highlighting the interplay between intrinsic and extrinsic motivations in promoting physical activity [59]. Numerous studies has identified a lack of exercise motivation as a critical factor contributing to insufficient physical activity among Chinese college students. This deficiency often stems from a lack of interest in sports and limited awareness of the health benefits of exercise [60] making it difficult for students to prioritize physical activity during their leisure time [61]. Without adequate motivation, students find it challenging to engage in exercise behavior. Numerous cross-sectional studies support that any type of exercise motivation is closely linked to increased participation in exercise behavior [62–64].

Personality traits have a wide range of predictive effects on motivation. Huang, Lee, and Chang [65] found that individuals with higher levels of Extraversion, Conscientiousness, and Openness tend to be more motivated to exercise, which in turn positively affects their overall quality of life. A study of elite athletes suggests that personality traits are significantly associated with motivation [66]. In addition, some studies have posted extraverted individuals may experience higher motivation due to their enjoyment of social interactions in exercise settings [67]. Conscientious individuals are likely to be motivated by the desire to achieve good health and accomplish related goals [68]. Based on this evidence and CAPS theory, exercise motivation may be a variable mediating personality traits and exercise behavior in college students.

As we hypothesized above, exercise self-efficacy and exercise motivation can play a mediating role in personality traits and exercise behavior of college students. However, when both exercise self-efficacy and exercise motivation are considered mediating roles, how do they relate to each other? According to SDT, the satisfaction of competence (a component of exercise self-efficacy) enhances autonomous motivation, leading to self-regulated behavior [69]. By integrating CAPS and SDT theories, we can establish a chain mediation pathway, the relationship between personality traits and exercise behavior may be mediated first through exercise self-efficacy and then exercise motivation.

**Fig. 1:**
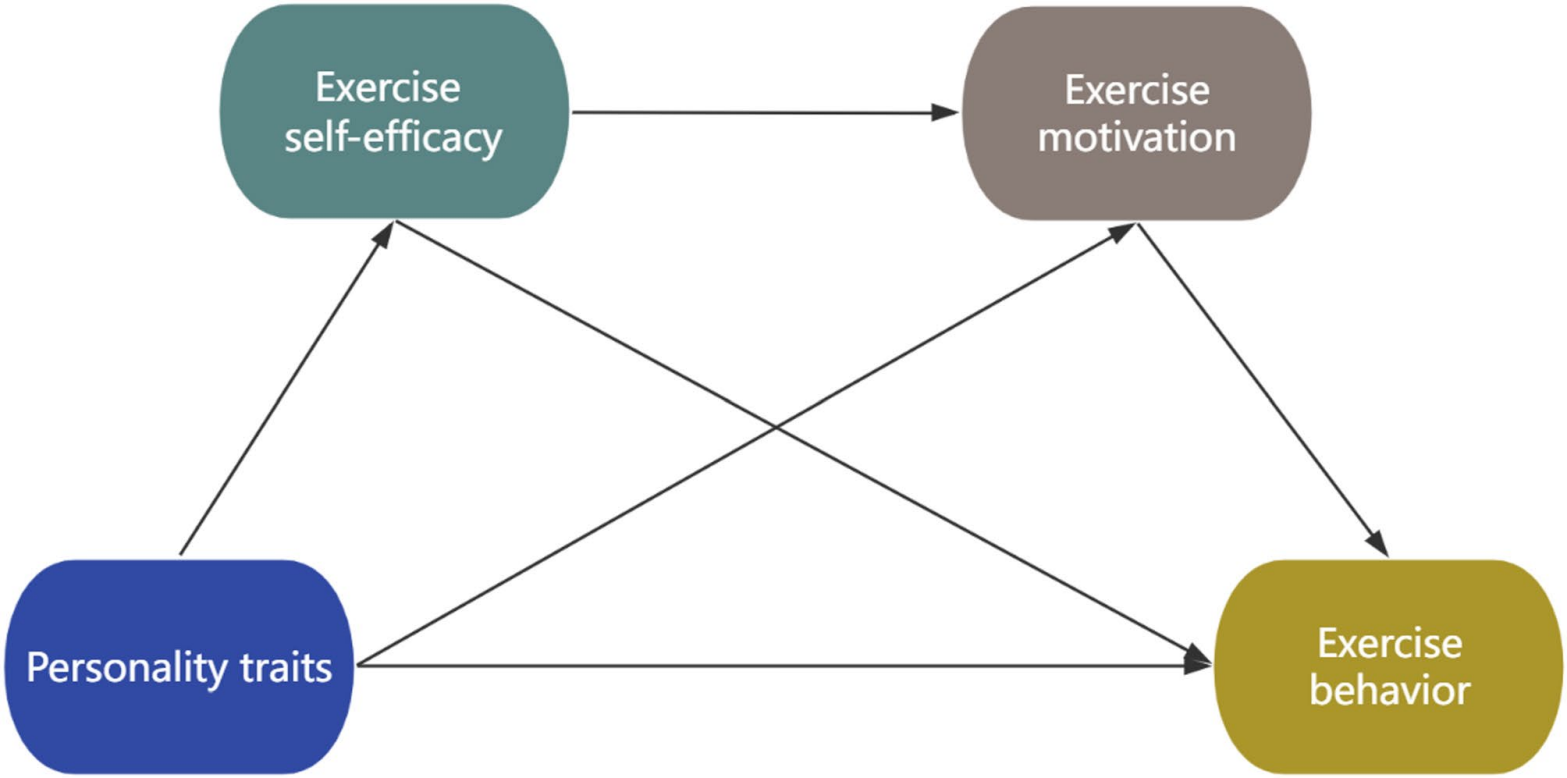
The proposed chain mediation model with four mediation paths tested based on different personality traits (Openness, Conscientiousness, Extraversion, Neuroticism)

Although some studies have confirmed that personality traits, exercise self-efficacy, exercise motivation, and exercise behavior are closely related, it remains unclear how they interact with each other. To our knowledge, this is the first study to construct a chain mediation model involving personality traits, exercise self-efficacy, exercise motivation, and exercise behavior. Considering the stability of personality traits, it is challenging to directly influence students’ exercise behavior by intervening their personality traits. However, it is feasible to achieve this goal by targeting the mediating factors. This study aims to clarify the internal mechanisms linking personality traits and exercise behavior, providing theoretical guidance for predicting and intervening in college students’ exercise behavior. Our practical goal is to promote their physical health by formulating effective health promotion strategies tailored to individual needs, thereby enhancing the level of physical exercise among the college student population. Based on previous research, we established a theoretical hypothesis model (shown in Fig. 1) and propose the following four hypotheses:

### H1

**Personality traits are significantly related to exercise behavior.**

H1(a): Openness is positively related to exercise behavior.

H1(b): Conscientiousness is positively related to exercise behavior.

H1(c): Extraversion is positively related to exercise behavior.

H1(d): Neuroticism is negatively related to exercise behavior.

H1(e): Agreeableness will show no significant association with exercise behavior.

### H2

**Exercise self-efficacy mediates the relationship between personality traits and exercise behavior.**

H2(a): Exercise self-efficacy mediates the positive relationship between Openness and exercise behavior.

H2(b): Exercise self-efficacy mediates the positive relationship between Conscientiousness and exercise behavior.

H2(c): Exercise self-efficacy mediates the positive relationship between Extraversion and exercise behavior.

H2(d): Exercise self-efficacy mediates the negative relationship between Neuroticism and exercise behavior.

### H3

**Exercise motivation mediates the relationship between personality traits and exercise behavior.**

H3(a): Exercise motivation mediates the positive association between Openness and exercise behavior.

H3(b): Exercise motivation mediates the positive association between Conscientiousness and exercise behavior.

H3(c): Exercise motivation mediates the positive association between Extraversion and exercise behavior.

H3(d): Exercise motivation mediates the negative association between Neuroticism and exercise behavior.

### H4

**Exercise self-efficacy and exercise motivation have a chain mediating effect between personality traits and exercise behavior.**

H4(a): The chain mediation of exercise self-efficacy and exercise motivation mediates the positive association between Openness and exercise behavior.

H4(b): The chain mediation of exercise self-efficacy and exercise motivation mediates the positive association between Conscientiousness and exercise behavior.

H4(c): The chain mediation of exercise self-efficacy and exercise motivation mediates the positive association between Extraversion and exercise behavior.

H4(d): The chain mediation of exercise self-efficacy and exercise motivation mediates the negative association between Neuroticism and exercise behavior.

## Materials and methods

### Participants and procedure

The study focuses on undergraduate students aged between 17 and 23 years. Students majoring in physical education were excluded due to the unique curriculum requirements of physical education majors [70]. To ensure a representative and diverse sample, we employed a two-stage sampling method. Specifically, we randomly selected five universities in Wuhan that varied in geographic location and academic reputation. Within each selected university, we further conducted cluster sampling by selecting non-physical education major classes to participate in the survey.

Data collection was carried out from October 2023 to November 2023 through class group chats. Specifically, we informed the instructors about the purpose and methodology of the survey. After providing their consent, the link to the electronic questionnaire was shared in the class groups for the students to complete. At baseline (Time 1; T1), the research purpose was explained to 512 students, who were assured of the strict confidentiality of their responses. The participants were then asked to complete demographic information, personality traits questionnaires, and exercise self-efficacy questionnaires. Four weeks later (Time 2; T2), during a class meeting, all 512 students completed exercise motivation and exercise behavior questionnaires. After filtering, 29 invalid questionnaires were excluded due to incomplete responses and uniform answers (e.g., all responses were rated as 5), and 483 valid questionnaires were recovered, for a retention rate of 94.34%.

In this study, we clearly informed all participants that they could withdraw at any time if they chose to do so. As a token of appreciation, all students who completed the second questionnaire received a chocolate bar.

### Instruments

#### Personality traits scale

The Chinese Big Five Personality Inventory Brief Version (CBF-PI-B) was developed by Wang to assess the Big Five personality traits in Chinese populations [71] based on the NEO Personality Inventory created by Costa and McCrae [26]. The CBF-PI-B is designed to align with the cultural and linguistic habits of the Chinese population and has been validated accordingly [72, 73]. The CBF-PI-B is a self-reported measure comprising 40 items covering five different traits, including Openness (e.g., “I have a great curiosity about many things”), Conscientiousness (e.g., “Once I have set a goal, I will work hard to achieve it”), Extraversion (e.g., “I like social and recreational gatherings”), Agreeableness (e.g., “I often feel sorry for those who have suffered misfortune”), and Neuroticism (e.g., “I always worry that something bad is going to happen”). For this study, we adapted the CBF-PI-B by selecting 25 items, each using a 5-point Likert scale ranging from 1 (“strongly disagree”) to 5 (“strongly agree”). The adapted scale demonstrated good internal consistency for each subscale: Openness (α = 0.869), Conscientiousness (α = 0.878), Extraversion (α = 0.924), Agreeableness (α = 0.907), and Neuroticism (α = 0.908). The KMO was 0.885, indicating sampling adequacy for factor analysis. In addition, the confirmatory factor analysis results further confirmed the validity of the adapted scale, with χ2/df = 1.794, RMSEA = 0.041, SRMR = 0.0341, IFI = 0.971, TLI = 0.967, CFI = 0.971, and GFI = 0.925. These results collectively indicate a strong fit to the proposed factor structure, supporting the reliability and validity of the adapted CBF-PI-B for use in this study.

#### Exercise self-efficacy scale

The Multidimensional Exercise Self-Efficacy (MESE) scale was created to assess students’ leisure-time exercise self-efficacy [74]. Previous studies have demonstrated that the MESE is effective in measuring the exercise self-efficacy across different populations [75, 76]. The MESE consists of 9 items categorized into three dimensions: task (e.g., “I have a belief in performing all of the required movements”), coping (e.g., “I have a belief in exercising when I lack energy”), and schedule (e.g., “I have a belief in arranging my time to include regular exercise”). Each item is assessed on a 5-point Likert scale ranging from 1 (“strongly disagree”) to 5 (“strongly agree”). The total Cronbach’s alpha for exercise self-efficacy scale was 0.816 (sub-scale alphas: task = 0.887, coping = 0.872, schedule = 0.888), and the KMO was 0.776. Besides, confirmatory factor analysis revealed χ2/df = 3.4, RMSEA = 0.071, SRMR = 0.0332, IFI = 0.977, TLI = 0.966, CFI = 0.977, and GFI = 0.963, supporting the scale’s reliability and validity.

#### Exercise motivation scale

Exercise motivation was assessed using the Chinese version of Motives for Physical Activities Measure‑Revised [56] Chen and colleagues reduced the number of items in the original MPAM-R [58] from 30 to 15 and translated the scale to align with the cultural context of Chinese. Other scholars have subsequently proven its reliability in measuring Chinese college students’ exercise motivation [77, 78]. The scale comprises 15 items categorized into five dimensions: enjoyment (e.g., “I want exercising to be pleasant”), ability (e.g., “I want to gain new sports skills”), social (e.g., “I want to meet new people”), appearance (e.g., “I want to improve my figure”), and health (e.g., “I want to get healthier”). Each item is answered on a five-point Likert-type scale, from 1 (“strongly disagree”) to 5 (“strongly agree”). The total Cronbach’s alpha for exercise motivation scale was 0.845 (sub-scale alphas: enjoyment = 0.856, ability = 0.879, social = 0.813, appearance = 0.92, health = 0.876), and the KMO was 0.745. Furthermore, confirmatory factor analysis results indicated a good fit for the scale, with χ2/df = 2.91, RMSEA = 0.063, SRMR = 0.032, IFI = 0.965, TLI = 0.953, CFI = 0.964, and GFI = 0.943.

#### Exercise behavior scale

The Physical Activity Rating Scale-3 (PAR-3) was created to evaluate students’ exercise behavior [79]. The PAR-3 comprises 3 items categorized according to intensity, duration, and frequency of exercise activity. Participants rated each item on a 5-point Likert scale. The PAR-3 scale combines three item scores into a total score, computed using the following formula: Intensity × (Duration − 1) × Frequency, and the score ranging is 0 to 100. Scores equal to or less than 19 were categorized as light, scores between 20 and 42 were categorized as moderate, and scores equal to or greater than 43 were categorized as vigorous. Nevertheless, our study, according to Xia, Huang and Liu, expanded the original three levels into four levels by introducing a category for basically no exercise (scores less than or equal to 4) below the light level [80]. This modification, supported by other authors [81] aims to distinguish students who do not engage in any exercise. The Cronbach’s alpha was 0.838, and the KMO was 0.716 for this study.

### Statistical analyses

In this study, all the data were analyzed using IBM SPSS 26.0 and AMOS 28.0. After all the questionnaires were collected, the data were subjected to the following steps: (1) Harman’s one-factor test was conducted on all the indicators using SPSS 26.0. (2) Exploratory factor analysis was performed for each scale using SPSS 26.0. (3) Cronbach’s alpha test was carried out for each scale using SPSS 26.0. (4) Confirmatory factor analysis was performed for all scales (excluding PAR-3) using AMOS 28.0. (5) The scores for exercise self-efficacy and exercise motivation were calculated by summing the scores of each dimension and then dividing by the number of dimensions. This approach ensured that composite scores accurately represented the overall levels of exercise self-efficacy and exercise motivation among participants [82] facilitating subsequent chain mediation model analyses [83]. (6) Descriptive statistics were analyzed using SPSS 26.0, and differences in demographic characteristics were examined by ANOVA and T-test. (7) Pearson’s R was utilized to explore the relationships between Openness, Conscientiousness, Extraversion, Agreeableness, Neuroticism, exercise self-efficacy, exercise motivation, and exercise behavior. (8) The Process V4.2 macro [83] in SPSS was used to test the chain mediating effects of exercise self-efficacy and exercise motivation between personality traits and exercise behavior in SPSS 26.0. For this study, the confidence interval was set at 95% and the significance threshold at *p* < 0.05. And, the correlation coefficient standards used in this study are: 0.10–0.29 indicates a small correlation, 0.30–0.49 indicates a medium correlation, and ≥ 0.50 indicates a large correlation [84].

## Results

### Common method bias test

The research data were gathered through students’ self-assessments. To check for common method bias in the data, Harman’s one-factor method test was conducted. The results of the exploratory factor analysis revealed 14 factors with initial eigenvalues greater than 1. Furthermore, the maximum factor variance explained was 22.811%, which was less than the threshold criterion of 40% [85]. This indicates that there is no issue of common method bias in the data for this study.

### Descriptive statistics and differences

A total of 483 participants (mean age = 19.71 ± 1.31) were included in this study. As shown in Tables 1, 212 participants (43.9%) were male, and 271 (56.1%) were female. Regarding gender, no significant differences were observed in Openness, Conscientiousness, Extraversion, Agreeableness, Neuroticism, exercise self-efficacy, and exercise behavior. However, a significant difference was detected in exercise motivation (*p* = 0.037 < 0.05), where males exhibited higher scores than females. Furthermore, no significant differences were identified in personality traits, exercise self-efficacy, exercise motivation, and exercise behavior in terms of age.

Regarding data distribution, the absolute values of skewness for all variables, except for exercise behavior, were less than 2, and the absolute values of kurtosis were less than 3, suggesting that the data were approximately normally distributed [86]. After applying a square root transformation to exercise behavior, its skewness and kurtosis also met the criteria for normality. These indicated that the data were suitable for subsequent correlation and chain mediation analysis.

**Table 1:** Descriptive statistics and statistical differences

			O	C	E	A	*N*	ESE	EM	EB (1)	EB (2)
Gender	*N*	%	Mean ± SD	Mean ± SD	Mean ± SD	Mean ± SD	Mean ± SD	Mean ± SD	Mean ± SD	Mean ± SD	Mean ± SD
Male	212	43.9%	3.01 ± 0.83	3.18 ± 0.81	3.17 ± 0.76	3.17 ± 0.84	2.77 ± 0.76	2.98 ± 0.53	3.17 ± 0.46*	14.41 ± 16.94	3.34 ± 1.8
Female	271	56.1%	2.87 ± 0.78	3.16 ± 0.82	3.09 ± 0.77	3.08 ± 0.8	2.71 ± 0.77	2.9 ± 0.52	3.08 ± 0.46*	12.55 ± 13.68	3.16 ± 1.6
Age											
17	13	2.7%	2.74 ± 0.66	3.09 ± 0.7	3.25 ± 0.86	3 ± 0.68	2.62 ± 0.56	3.09 ± 0.45	3.17 ± 0.4	12.23 ± 12.72	3.17 ± 1.53
18	85	17.6%	2.97 ± 0.76	3.26 ± 0.83	3.11 ± 0.83	3.03 ± 0.81	2.66 ± 0.69	2.99 ± 0.51	3.14 ± 0.41	15.59 ± 16.33	3.52 ± 1.8
19	120	24.8%	2.85 ± 0.7	3.17 ± 0.69	3.09 ± 0.74	3.12 ± 0.8	2.75 ± 0.81	2.95 ± 0.53	3.13 ± 0.45	12.68 ± 14.13	3.18 ± 1.61
20	122	25.3%	2.99 ± 0.92	3.14 ± 0.92	3.21 ± 0.76	3.22 ± 0.85	2.74 ± 0.81	2.93 ± 0.53	3.11 ± 0.5	14.41 ± 17.29	3.31 ± 1.86
21	108	22.4%	2.98 ± 0.87	3.18 ± 0.81	3.09 ± 0.74	3.07 ± 0.81	2.74 ± 0.78	2.92 ± 0.55	3.11 ± 0.5	12.84 ± 14.18	3.21 ± 1.6
22	26	5.4%	2.78 ± 0.65	2.95 ± 0.81	2.93 ± 0.73	3.1 ± 0.79	2.92 ± 0.69	2.72 ± 0.48	3.06 ± 0.46	9.31 ± 12.75	2.69 ± 1.47
23	9	1.9%	2.91 ± 0.66	3.2 ± 0.99	3.22 ± 0.75	3.53 ± 0.98	2.73 ± 0.42	3.05 ± 0.5	3.07 ± 0.29	7 ± 4.42	2.55 ± 0.76
Skewness			0.304	0.204	-0.015	0.157	0.597	-0.204	0.149	2.28	1.339
Kurtosis			-0.191	-0.025	0.054	-0.578	-0.124	0.088	0.2	5.783	1.297

*P < 0.05, **P < 0.01, ***P < 0.001, O = Openness, C = Conscientiousness, E = Extraversion, A = Agreeableness, *N* = Neuroticism, ESE = Exercise Self-efficacy, EM = Exercise Motivation, EB = Exercise Behavior, EB (1) indicates before Square Root Transformation, EB (2) indicates after Square Root Transformation

### Correlation analysis

According to the correlation results presented in Table 2, Openness was positively correlated with exercise motivation and exercise behavior. Conscientiousness exhibited positive correlations with exercise self-efficacy, exercise motivation, and exercise behavior. Extraversion was positively correlated with exercise self-efficacy, exercise motivation, and exercise behavior. Agreeableness was not significantly correlated with exercise self‑efficacy, exercise motivation, or exercise behavior. Neuroticism showed negative correlations with exercise self-efficacy, exercise motivation, and exercise behavior. Therefore, these results support our hypotheses H1 that Openness, Conscientiousness, and Extraversion are positively related to exercise behavior, while Neuroticism is negatively related to exercise behavior. Accordingly, because Agreeableness showed no significant associations with exercise selfefficacy, exercise motivation, or exercise behavior, it was not included in the subsequent mediation analyses.

**Table 2:** Correlations for all variables

Variables	Mean ± SD	O	C	E	A	*N*	ESE	EM	EB
O	2.93 ± 0.8	--							
C	3.17 ± 0.81	0.209**	--						
E	3.12 ± 0.76	0.211**	0.316**	--					
A	3.12 ± 0.82	0.052	0.183**	0.146**	--				
*N*	2.74 ± 0.76	− 0.110*	− 0.268**	− 0.286**	− 0.121**	--			
ESE	2.94 ± 0.53	0.064	0.277**	0.332**	0.086	− 0.249**	--		
EM	3.12 ± 0.46	0.248**	0.347**	0.392**	0.064	− 0.271**	0.464**	--	
EB	3.24 ± 1.69	0.221**	0.269**	0.413**	0.08	− 0.313**	0.504**	0.493**	--

*P < 0.05, **P < 0.01, O = Openness, C = Conscientiousness, E = Extraversion, A = Agreeableness, *N* = Neuroticism, ESE = Exercise Self-efficacy, EM = Exercise Motivation, EB = Exercise Behavior, The value of EB has been square root transformed

### The chain mediating effect test

Following the instructions of the analytical approach for testing the chain mediation model [83, 87] a chain mediation analysis was performed using Process Model 6 with 5000 bootstrap samples. Gender and age were included as control variables, as both have been shown in previous research to influence exercise self-efficacy, motivation, and behavior [88, 89]. Exercise self-efficacy and exercise motivation were specified as mediators, with exercise behavior as the outcome variable. The personality traits Openness, Conscientiousness, Extraversion, and Neuroticism were entered as predictor variables in four separate chain mediation models.

**Fig. 2:**
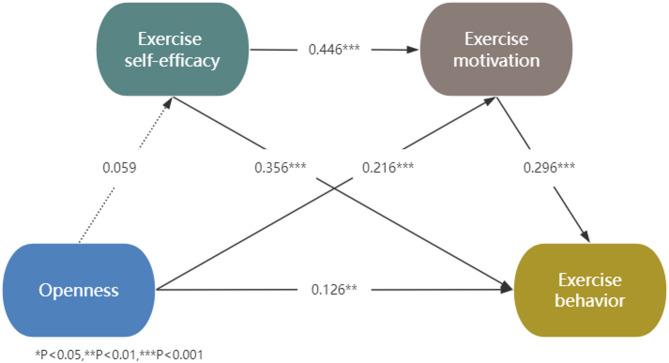
Chain mediation model of openness on exercise behavior

In the Openness model (see Fig. 2), Openness significantly predicted exercise motivation and exercise behavior but did not significantly predict exercise self-efficacy. Furthermore, exercise motivation significantly predicted exercise behavior. Mediation analysis revealed that exercise motivation mediated the positive relationship between Openness and exercise behavior. However, since Openness was not significantly associated with exercise self-efficacy, neither the mediation effect of exercise self-efficacy nor the chain mediation effect through exercise self-efficacy and exercise motivation was supported. Additionally, Table 3 showed that Openness was directly related to exercise behavior (accounting for 57.70% of the total effect) and indirectly related to exercise behavior through exercise motivation (accounting for 29.28% of the total effect), with no contribution from exercise self-efficacy or chain mediation (the 95% confidence interval includes zero). For more detailed information on the chain mediation results, including model fit indices and effect sizes, please refer to Supplementary Tables 1–4.

**Fig. 3:**
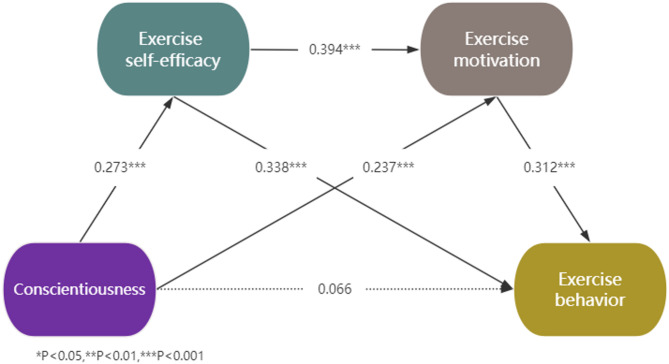
Chain mediation model of conscientiousness on exercise behavior

In the Conscientiousness model (see Fig. 3), Conscientiousness significantly predicted both exercise self-efficacy and exercise motivation, while exercise self-efficacy and exercise motivation significantly predicted exercise behavior. Mediation analysis revealed that exercise self-efficacy mediated the positive relationship between Conscientiousness and exercise behavior, supporting our hypothesis that exercise self-efficacy serves as a mediator. Similarly, exercise motivation acted as a mediator in this positive relationship, confirming our hypothesis regarding the mediating role of exercise motivation. Furthermore, a significant chain mediation effect was observed, indicating that Conscientiousness is associated with exercise behavior through the sequential mediators exercise self-efficacy and exercise motivation, thereby supporting our hypothesis of chain mediation. Additionally, Table 3 showed that Conscientiousness was indirectly related to exercise behavior through exercise self-efficacy (accounting for 34.72% of the total effect), exercise motivation (accounting for 27.85% of the total effect), and the chain mediation of exercise self-efficacy and exercise motivation (accounting for 12.66% of the total effect). Moreover, Conscientiousness was not directly related to exercise behavior (the 95% confidence interval includes zero). These findings demonstrate that the relationship between Conscientiousness and exercise behavior is fully mediated by exercise self-efficacy and exercise motivation.

**Fig. 4:**
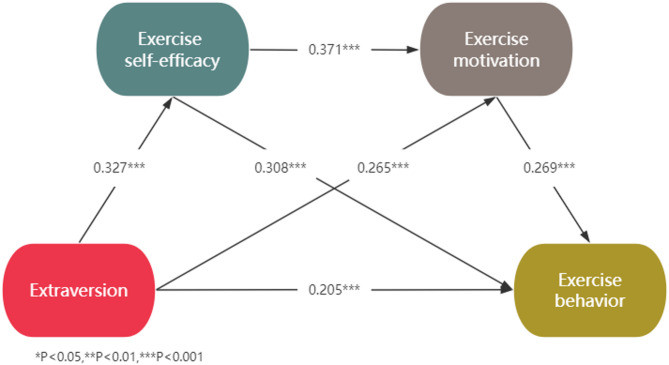
Chain mediation model of extraversion on exercise behavior

In the Extraversion model (see Fig. 4), Extraversion significantly predicted exercise self-efficacy, exercise motivation, and exercise behavior, while both exercise self-efficacy and exercise motivation significantly predicted exercise behavior. Mediation analysis revealed that exercise self-efficacy mediated the positive relationship between Extraversion and exercise behavior, supporting our hypothesis that exercise self-efficacy serves as a mediator, and exercise motivation also acted as a mediator in this relationship, confirming our hypothesis regarding the mediating role of exercise motivation. Furthermore, a significant chain mediation effect was observed, indicating that Extraversion was associated with exercise behavior through the sequential mediators exercise self-efficacy and exercise motivation, supporting our hypothesis of a chain mediation. Additionally, Table 3 showed that Extraversion was directly related to exercise behavior (accounting for 50.05% of the total effect) and indirectly related to exercise behavior through exercise self-efficacy (accounting for 24.59% of the total effect), exercise motivation (accounting for 17.34% of the total effect), and the chain mediation of exercise self-efficacy and exercise motivation (accounting for 7.90% of the total effect). These findings demonstrate that the relationship between Extraversion and exercise behavior is partially mediated by exercise self-efficacy and exercise motivation.

**Fig. 5:**
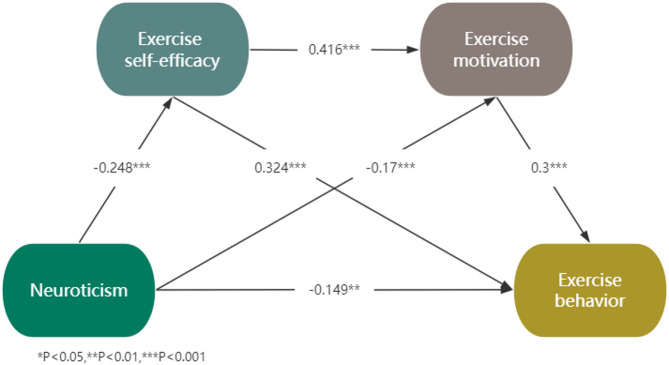
Chain mediation model of neuroticism on exercise behavior

In the Neuroticism model (see Fig. 5), Neuroticism significantly and negatively predicted exercise self-efficacy, exercise motivation, and exercise behavior, while both exercise self-efficacy and exercise motivation significantly predicted exercise behavior. Mediation analysis revealed that exercise self-efficacy mediated the negative relationship between Neuroticism and exercise behavior, supporting our hypothesis that exercise self-efficacy serves as a mediator, and exercise motivation also acted as a mediator in this relationship, confirming our hypothesis regarding the mediating role of exercise motivation. Furthermore, a significant chain mediation effect was observed, indicating that Neuroticism was associated with exercise behavior through the sequential mediators exercise self-efficacy and exercise motivation, supporting our hypothesis of a chain mediation. In addition, Table 3 showed that Neuroticism was directly associated with exercise behavior (accounting for 47.83% of the total effect) and indirectly associated with exercise behavior through exercise self-efficacy (accounting for 25.87% of the total effect), exercise motivation (accounting for 16.33% of the total effect), and the chain mediation of exercise self-efficacy and exercise motivation (accounting for 9.97% of the total effect). These findings demonstrate that the relationship between Neuroticism and exercise behavior is partially mediated by exercise self-efficacy and exercise motivation.

**Table 3:** Chain mediated analysis pathways for the four personality traits (Bootstrap)

Model	Pathways	Effect	Boot SE	Boot LLCI	Boot ULCI	Proportion of Total effect
Openness	Total effect (C)	0.461	0.094	0.277	0.645	
	Direct effect (C’)	0.266	0.08	0.108	0.423	57.70%
	Total indirect effect	0.195	0.054	0.09	0.303	42.30%
	X ->ESE->EB	0.044	0.034	-0.021	0.113	
	X ->EM ->EB	0.135	0.032	0.077	0.2	29.28%
	X ->ESE ->EM ->EB	0.016	0.013	-0.007	0.044	
Conscientiousness	Total effect (C)	0.553	0.091	0.373	0.732	
	Direct effect (C’)	0.137	0.083	-0.027	0.3	
	Total indirect effect	0.416	0.06	0.302	0.538	75.23%
	X ->ESE->EB	0.192	0.04	0.119	0.276	34.72%
	X ->EM ->EB	0.154	0.034	0.092	0.228	27.85%
	X ->ESE ->EM ->EB	0.07	0.017	0.04	0.105	12.66%
Extraversion	Total effect (C)	0.911	0.092	0.73	1.092	
	Direct effect (C’)	0.456	0.089	0.281	0.63	50.05%
	Total indirect effect	0.455	0.057	0.346	0.568	49.95%
	X ->ESE->EB	0.224	0.041	0.145	0.308	24.59%
	X ->EM ->EB	0.158	0.037	0.092	0.235	17.34%
	X ->ESE ->EM ->EB	0.072	0.019	0.04	0.113	7.90%
Neuroticism	Total effect (C)	-0.692	0.096	-0.88	-0.503	
	Direct effect (C’)	-0.331	0.085	-0.499	-0.163	47.83%
	Total indirect effect	-0.361	0.054	-0.47	-0.259	52.17%
	X ->ESE->EB	-0.179	0.037	-0.255	-0.11	25.87%
	X ->EM ->EB	-0.113	0.033	-0.185	-0.056	16.33%
	X ->ESE ->EM ->EB	-0.069	0.017	-0.105	-0.041	9.97%

X = Predictor Variables (Openness, Conscientiousness, Extraversion, Neuroticism), ESE = Exercise self-efficacy, EM = Exercise motivation, EB = Exercise behavior. LLCI to ULCI not cross 0, the mediating effect is significant

## Discussion

This study employed a chain mediation model to examine the associations between personality traits and exercise behavior among Chinese college students. By integrating the CAPS and SDT frameworks, we explored the potential mechanisms through which personality traits are related to exercise behavior. Our findings not only corroborate previous research on the relationship between personality traits and exercise behavior but also provide deeper insights into how exercise self-efficacy and exercise motivation serve as mediators in this relationship, thereby enriching our understanding of this domain.

### Gender differences

These results revealed significant gender differences in exercise motivation, with males scoring higher than females. This discrepancy may be explained by sociocultural factors and gender role expectations. Specifically, physical activities are often seen as symbols of strength and competition [90] and males typically receive more encouragement and social recognition when engaging in these activities. This support not only enhances males’ motivation to improve their self-worth through exercise but also reinforces their sense of identity and social status [91]. Additionally, the extensive media coverage and positive reporting on male sports activities may subconsciously boost males’ enthusiasm for participating in exercise [92]. Conversely, women might encounter traditional beliefs of norms typically glorify a delicate, feminine image, which conflict with the emphasis on strength, competition, and adventurousness in physical activities. resulting in lower level of exercise motivation [93, 94]. Moreover, societal expectations for females to fulfill family and caregiving roles often limit their time and energy for physical activity [94].

### The relationship between personality traits and exercise behavior

Consistent with prior studies, our findings indicate that certain personality traits are closely associated with exercise behavior. Specifically, Openness, Conscientiousness, and Extraversion were positively related to exercise behavior (*r* = 0.221, 0.269, 0.413), whereas Neuroticism was negatively associated (*r* = -0.313) [20, 29, 33, 95]. The results of the correlation analyses indicate that Extraversion exhibits a medium correlation with exercise behavior, followed by Conscientiousness and Openness, which show small positive correlations. In contrast, Neuroticism has a medium negative relation to exercise behavior. The medium correlation between Extraversion and exercise behavior reflects the direct enhancement of exercise behavior driven by Extraversion’s high-energy traits [95]. Conversely, the lower correlations observed for Conscientiousness and Openness imply that these traits contribute to exercise behavior in more subtle and context-dependent ways, possibly through complex relationships involving psychological factors such as exercise self-efficacy and exercise motivation [33]. As hypothesized, Agreeableness did not significantly relate to exercise behavior (*r* = 0.08, *p* = 0.079), this finding aligns with previous research demonstrating weak associations between Agreeableness and health-related behaviors [33–35] possibly due to its limited motivational relevance in autonomous goal pursuits such as exercise. Overall, these results support the predictive role of personality traits in health behavior choices and underscores the significance of emotional experiences and regulation in influencing individual behaviors [23].

### The mediating role of exercise self-efficacy and exercise motivation

These findings confirm previous research on the mediating role of exercise self-efficacy and exercise motivation in exercise behavior, with varying effects across different personality traits [51, 65, 95] and further deepen our understanding of these differential mediation effects. Specially, the association between Conscientiousness and exercise behavior is mainly mediated through exercise self-efficacy. Conscientious individuals, characterized by their self-discipline and planning, are more likely to have greater confidence in their exercise abilities [53] thereby participating in physical activities. Additionally, exercise motivation also plays an important role, as Conscientious individuals tend to set clear training programs and exercise goals (to improve ability or maintain health) and commit to achieving these goals [25, 29].

Similarly, Extraversion is linked to exercise behavior through both exercise self-efficacy and exercise motivation, with exercise self-efficacy playing a more significant role. The vitality and sociable traits of extraverted individuals are associated with their confidence in their exercise abilities, making them more likely to overcome obstacles and thereby engage more actively in exercise behaviors [42]. While the effect of exercise motivation is less pronounced than that of exercise self-efficacy, it still plays an important role. Extraverted individuals’ social needs and preference for external stimuli are associated with an increase in their exercise motivation, which subsequently influences their exercise behavior [20, 53].

In contrast, the negative association between Neuroticism and exercise behavior is mediated by exercise self-efficacy and exercise motivation. Individuals with high Neuroticism tend to experience emotional instability and anxiety, which diminishes their confidence in their exercise abilities, thereby reducing their likelihood of engaging in exercise [96]. Moreover, negative emotions and pessimistic expectations may suppress their exercise motivation of individuals with high Neuroticism, further diminishing their willingness to participate in physical activities [31]. However, the effect through exercise self-efficacy was notably stronger than that through exercise motivation. This suggests that emotional instability and a lack of self-confidence are associated with a more substantial impact on reducing exercise participation than the suppression of motivation alone.

Unlike other personality traits, the positive relationship between Openness and exercise behavior is mediated solely through exercise motivation. This may come from the fact that open-minded individuals’ desire and curiosity for new experiences fuels their intrinsic motivation to actively participate in a variety of novel physical activities [97, 98]. exercise self-efficacy is not significant in the pathway of Openness, possibly because individuals high in Openness are more focused on the novel experiences that behaviors bring, rather than on their ability to successfully engage in those behaviors [32]. Meanwhile, Wang et al. (2023) also pointed out that the relationship between Openness and self-efficacy is weak [53].

### The chain mediating effect of exercise self-efficacy and exercise motivation

This study revealed a significant chain mediation pathway involving exercise self-efficacy and exercise motivation in the associations between specific personality traits and exercise behavior. This finding is theoretically grounded in the CAPS theory, which suggests that personality traits interact dynamically with situational contexts through cognitive-affective units such as self-efficacy beliefs and motivational orientations [99].

Specifically, exercise self-efficacy represents individuals’ cognitive-affective appraisal of their perceived capability to cope with exercise-related tasks [41]. Exercise motivation, which follows this cognitive appraisal, involves motivational tendencies towards exercise, particularly autonomous forms of motivation [58]. Consistent with Bandura’s SCT, individuals who perceive themselves as efficacious are more likely to develop autonomous and intrinsically regulated motivations [41]. SDT further supports this perspective, emphasizing that higher perceived competence, a construct closely related to self-efficacy, is essential in fostering intrinsic motivation and ultimately enhancing sustained engagement in exercise [67, 69].

In the present findings, Conscientiousness, Extraversion, and Neuroticism demonstrated significant indirect relationships with exercise behavior through the sequential pathways of exercise self-efficacy and exercise motivation. Notably, the relationship between Conscientiousness and exercise behavior was entirely mediated by these two factors. Conscientious individuals, characterized by traits such as self-discipline and diligence, typically report higher levels of self-efficacy and intrinsic motivation toward exercise, which are subsequently associated with greater exercise engagement [100–102]. This fully mediated relationship underscores the critical role cognitive-affective processes play in linking conscientious traits to exercise behavior.

Extraversion exhibited both direct and indirect associations with exercise behavior, suggesting partial mediation through exercise self-efficacy and exercise motivation. Extraverted individuals generally possess high levels of social confidence and energy, contributing to enhanced self-efficacy beliefs and autonomous motivation, both positively correlated with increased exercise behavior [33, 67]. Similarly, Neuroticism showed negative associations, indicating that individuals with higher emotional instability may experience reduced exercise self-efficacy and exercise motivation, thereby correlating with decreased exercise participation [100, 101].

Collectively, these results highlight the importance of both cognitive-affective appraisals and motivational orientations in understanding how personality traits relate to exercise behavior. The integration of CAPS, SCT, and SDT provides a robust theoretical framework elucidating these complex pathways.

### Practical implications

The findings of this study suggest potential practical implications for promoting exercise behavior among college students. Given the relative stability of personality traits, interventions should focus on modifiable psychological factors, such as exercise self-efficacy and motivation. Tailored strategies that align with individual personality profiles may enhance intervention effectiveness. For students high in Openness, offering diverse and novel exercise opportunities may stimulate intrinsic motivation. Conscientious individuals may benefit from structured goal-setting, skill development, and positive feedback to strengthen self-efficacy. Extraverts may respond well to group-based programs that incorporate social interaction, while personalized, achievable tasks may reduce anxiety and enhance motivation among those high in Neuroticism. Furthermore, enhancing self-efficacy through successful exercise experiences and fostering supportive exercise environments can further meet students’ needs for competence and autonomy, thereby promoting sustained participation in physical activities. Additionally, by recognizing the unique barriers faced by women in physical activity and actively promoting inclusive and supportive environments, exercise motivation and participation among female students can be significantly improved. However, it should be noted that these proposed strategies are theoretical in nature and have not yet been empirically validated. Therefore, future intervention studies are needed to test the real-world applicability and impact of these personality-informed approaches.

### Limitations and prospects

While this study enriches the understanding of the inherent connections among personality traits, exercise self-efficacy, exercise motivation, and exercise behavior, it is not without limitations. Firstly, the assessment of exercise behavior was based on self-reported data, which may be influenced by social desirability and personal biases, potentially resulting in inaccuracies. Secondly, the examination of demographic variables such as age and gender was constrained, lacking an in-depth exploration of how these variables may moderate the relationships among personality traits, exercise self-efficacy, exercise motivation, and exercise behavior. Thirdly, the study exclusively focused on the mediating roles of self-efficacy and motivation, overlooking other real-world influencing factors such as environmental variables, social support, and cultural norms, which may also significantly shape exercise behavior. Fourth, the personality scale employed might not fully capture the entire spectrum of personality traits, potentially affecting the study’s findings. Additionally, the aggregation of autonomous and controlled motivations into an integrated score may obscure the distinct mediation effects that different types of motivation might have on exercise behavior. Lastly, the cross-sectional design of this study precludes the establishment of temporal sequences among variables. Although significant associations between personality traits, exercise self-efficacy, exercise motivation, and exercise behavior were identified, these findings should be interpreted cautiously as correlations rather than causal relationships. To further clarify the directionality and temporal ordering of these mechanisms, future studies employing longitudinal or experimental designs are recommended. By addressing these limitations, future research can build upon the findings of this study and further refine the understanding of the mechanisms linking personality traits, exercise self-efficacy, exercise motivation, and exercise behavior.

## Conclusion

In conclusion, this study elucidates the mechanisms through which Chinese college students’ personality traits are associated with their exercise behavior, highlighting the varying degrees of mediating roles of exercise self-efficacy and exercise motivation. Notably, partial mediation through both self-efficacy and motivation was observed for Extraversion and Neuroticism. In contrast, the pathway for Openness was mediated solely by exercise motivation, with no significant contribution from self-efficacy. Meanwhile, the relationship between Conscientiousness and exercise behavior was fully mediated by both self-efficacy and motivation, indicating a unique and complete indirect effect. These results underscore the importance of exercise self-efficacy and exercise motivation in promoting exercise behavior among college students. Consequently, intervention measures should prioritize boosting students’ confidence and intrinsic motivation, developing personalized health promotion strategies to improve their physical health and prevent related health risks.

## Supplementary Information

Below is the link to the electronic supplementary material.

Supplementary Material 1

Supplementary Material 2

## Data Availability

The datasets used in this study are available from the corresponding author on reasonable request.

## Abbreviations

CAPS: Cognitive-Affective Personality System Theory
CBF-PI-B: Chinese Big Five Personality Inventory Brief Version
MESE: Multidimensional Exercise Self-Efficacy
PAR-3: Physical Activity Rating Scale-3
SCT: Social Cognitive Theory
SDT: Self-Determination Theory
