# Supplementary material for: The influence of personality traits on college students’ exercise behavior: a chain mediation model of exercise self-efficacy and exercise motivation
Source: BMC Psychol. 2025 Aug 4;13:864. doi: 10.1186/s40359-025-03220-y (PMC12323249; doi:10.1186/s40359-025-03220-y)
Supplement: Supplementary file 1 — Supplementary Material 1 [file 40359_2025_3220_MOESM1_ESM.docx]

Supplementary table

ST 1 Regression analysis of the Openness chain-mediated model

| Regression equation | |  | Fit index | | |  | Effect | |
| --- | --- | --- | --- | --- | --- | --- | --- | --- |
| Outcome variables | Predictive variables |  | R | R^2^ | f |  | β | t |
| EB | O |  | 0.24 | 0.057 | 9.716 |  | 0.219 | 4.914*** |
|  | Genders |  |  |  |  |  | -0.037 | -0.825 |
|  | Age |  |  |  |  |  | -0.086 | -1.947 |
| ESE | O |  | 0.126 | 0.016 | 2.582 |  | 0.059 | 1.291 |
|  | Genders |  |  |  |  |  | -0.073 | -1.61 |
|  | Age |  |  |  |  |  | -0.083 | -1.821 |
| EM | O |  | 0.515 | 0.265 | 43.19 |  | 0.216 | 5.491*** |
|  | ESE |  |  |  |  |  | 0.446 | 11.292*** |
|  | Genders |  |  |  |  |  | -0.042 | -1.073 |
|  | Age |  |  |  |  |  | -0.012 | -0.314 |
| EB | O |  | 0.597 | 0.356 | 52.825 |  | 0.126 | 3.311** |
|  | ESE |  |  |  |  |  | 0.356 | 8.549*** |
|  | EM |  |  |  |  |  | 0.296 | 6.905*** |
|  | Genders |  |  |  |  |  | 0.012 | 0.312 |
|  | Age |  |  |  |  |  | -0.042 | -1.15 |
| *P<0.05, **P<0.01, ***P<0.001, O = Openness, ESE = Exercise Self-efficacy, EM = Exercise Motivation, EB= Exercise Behavior, β = standard effect | | | | | | | | |

ST 2 Regression analysis of the Conscientiousness chain-mediated model

| Regression equation | |  | Fit index | | |  | Effect | |
| --- | --- | --- | --- | --- | --- | --- | --- | --- |
| Outcome variables | Predictive variables |  | R | R^2^ | f |  | β | t |
| EB | C |  | 0.283 | 0.08 | 13.911 |  | 0.265 | 6.051*** |
|  | Genders |  |  |  |  |  | -0.052 | -1.187 |
|  | Age |  |  |  |  |  | -0.072 | -1.635 |
| ESE | C |  | 0.295 | 0.087 | 15.189 |  | 0.273 | 6.246*** |
|  | Genders |  |  |  |  |  | -0.075 | -1.71 |
|  | Age |  |  |  |  |  | -0.07 | -1.593 |
| EM | C |  | 0.521 | 0.271 | 44.414 |  | 0.237 | 5.829*** |
|  | ESE |  |  |  |  |  | 0.394 | 9.631*** |
|  | Genders |  |  |  |  |  | -0.062 | -1.58 |
|  | Age |  |  |  |  |  | -0.003 | -0.085 |
| EB | C |  | 0.588 | 0.345 | 50.314 |  | 0.066 | 1.643 |
|  | ESE |  |  |  |  |  | 0.338 | 7.983*** |
|  | EM |  |  |  |  |  | 0.312 | 7.194*** |
|  | Genders |  |  |  |  |  | 0.002 | 0.046 |
|  | Age |  |  |  |  |  | -0.039 | -1.038 |
| *P<0.05, **P<0.01, ***P<0.001, C = Conscientiousness, ESE = Exercise Self-efficacy, EM = Exercise Motivation, EB= Exercise Behavior, β = standard effect | | | | | | | | |

ST 3 Regression analysis of the Extraversion chain-mediated model

| Regression equation | |  | Fit index | | |  | Effect | |
| --- | --- | --- | --- | --- | --- | --- | --- | --- |
| Outcome variables | Predictive variables |  | R | R^2^ | f |  | β | t |
| EB | E |  | 0.421 | 0.178 | 34.47 |  | 0.41 | 9.884*** |
|  | Genders |  |  |  |  |  | -0.034 | -0.824 |
|  | Age |  |  |  |  |  | -0.075 | -1.81 |
| ESE | E |  | 0.345 | 0.119 | 21.636 |  | 0.327 | 7.623*** |
|  | Genders |  |  |  |  |  | -0.061 | -1.426 |
|  | Age |  |  |  |  |  | -0.075 | -1.748 |
| EM | E |  | 0.531 | 0.282 | 46.872 |  | 0.265 | 6.454*** |
|  | ESE |  |  |  |  |  | 0.371 | 8.989*** |
|  | Genders |  |  |  |  |  | -0.053 | -1.363 |
|  | Age |  |  |  |  |  | -0.01 | -0.261 |
| EB | E |  | 0.613 | 0.376 | 57.499 |  | 0.205 | 5.134*** |
|  | ESE |  |  |  |  |  | 0.308 | 7.403*** |
|  | EM |  |  |  |  |  | 0.269 | 6.292*** |
|  | Genders |  |  |  |  |  | 0.005 | 0.138 |
|  | Age |  |  |  |  |  | -0.042 | -1.148 |
| *P<0.05, **P<0.01, ***P<0.001, E = Extraversion, ESE = Exercise Self-efficacy, EM = Exercise Motivation, EB= Exercise Behavior, β = standard effect | | | | | | | | |

ST 4 Regression analysis of the Neuroticism chain-mediated model

| Regression equation | |  | Fit index | | |  | Effect | |
| --- | --- | --- | --- | --- | --- | --- | --- | --- |
| Outcome variables | Predictive variables |  | R | R^2^ | f |  | β | t |
| EB | N |  | 0.327 | 0.107 | 19.095 |  | -0.312 | -7.212*** |
|  | Genders |  |  |  |  |  | -0.067 | -1.547 |
|  | Age |  |  |  |  |  | -0.068 | -1.561 |
| ESE | N |  | 0.272 | 0.074 | 12.748 |  | -0.248 | -5.638*** |
|  | Genders |  |  |  |  |  | -0.087 | -1.984* |
|  | Age |  |  |  |  |  | -0.069 | -1.568 |
| EM | N |  | 0.496 | 0.246 | 39.013 |  | -0.17 | -4.136*** |
|  | ESE |  |  |  |  |  | 0.416 | 10.091*** |
|  | Genders |  |  |  |  |  | -0.069 | -1.74 |
|  | Age |  |  |  |  |  | -0.003 | -0.082 |
| EB | N |  | 0.601 | 0.362 | 54.065 |  | -0.149 | -3.879*** |
|  | ESE |  |  |  |  |  | 0.324 | 7.748*** |
|  | EM |  |  |  |  |  | 0.3 | 7.124*** |
|  | Genders |  |  |  |  |  | -0.007 | -0.184 |
|  | Age |  |  |  |  |  | -0.036 | -0.967 |
| *P<0.05, **P<0.01, ***P<0.001, N = Neuroticism, ESE = Exercise Self-efficacy, EM = Exercise Motivation, EB= Exercise Behavior, β = standard effect | | | | | | | | |

ST 5 Reliability and validity tests

|  | **Cronbach's Alpha** | **KMO** | **Bartlett's Chi-Square (P-value)** | **Cumulative variance contribution rate** | **items** |
| --- | --- | --- | --- | --- | --- |
| Openness | 0.854 |  |  |  | 5 |
| Conscientiousness | 0.924 |  |  |  | 5 |
| Extraversion | 0.878 |  |  |  | 5 |
| Agreeableness | 0.907 |  |  |  | 5 |
| Neuroticism | 0.908 |  |  |  | 5 |
| **Personality** | **0.786** | **0.885** | **7449.231***** | **71.521** | **25** |
| Enjoy | 0.856 |  |  |  | 3 |
| Socal | 0.813 |  |  |  | 3 |
| health | 0.876 |  |  |  | 3 |
| appearance | 0.92 |  |  |  | 3 |
| ability | 0.879 |  |  |  | 3 |
| **EM** | **0.845** | **0.745** | **4339.67***** | **79.974** | **15** |
| Task | 0.887 |  |  |  | 3 |
| Coping | 0.872 |  |  |  | 3 |
| Schedule | 0.888 |  |  |  | 3 |
| **ESE** | **0.816** | **0.776** | **2547.452***** | **81.245** | **9** |
| **EB** | **0.838** | **0.716** | **591.894***** | **75.729** | **3** |
| **All** | **0.881** | **0.865** | **16319.644***** | **76.863** | **52** |
| ESE = Exercise Self-efficacy, EM = Exercise Motivation, EB= Exercise Behavior | | | | | |
